# Integrated transcriptome and microRNA sequencing analyses reveal gene responses in poplar leaves infected by the novel pathogen bean common mosaic virus (BCMV)

**DOI:** 10.3389/fpls.2023.1163232

**Published:** 2023-06-15

**Authors:** Li Wang, Weixi Zhang, Wanna Shen, Min Li, Yuchen Fu, Zheng Li, Jinxin Li, Huixiang Liu, Xiaohua Su, Bingyu Zhang, Jiaping Zhao

**Affiliations:** ^1^ State Key Laboratory of Tree Genetics and Breeding, Research Institute of Forestry, Chinese Academy of Forestry, Beijing, China; ^2^ State Key Laboratory of Tree Genetics and Breeding, Institute of Ecological Conservation and Restoration, Chinese Academy of Forestry, Beijing, China; ^3^ Shandong Research Center for Forestry Harmful Biological Control Engineering and Technology, College of Plant Protection, Shandong Agricultural University, Taian, China

**Keywords:** *Populus alba* var. *pyramidalis*, mRNA-seq, miRNA-seq, bean common mosaic virus, flavonoids biosynthesis, photosynthesis, miR156/SPL module

## Abstract

Recently, a novel poplar mosaic disease caused by bean common mosaic virus (BCMV) was investigated in *Populus alba* var. *pyramidalis* in China. Symptom characteristics, physiological performance of the host, histopathology, genome sequences and vectors, and gene regulation at the transcriptional and posttranscriptional levels were analyzed and RT−qPCR (quantitative reverse transcription PCR) validation of expression was performed in our experiments. In this work, the mechanisms by which the BCMV pathogen impacts physiological performance and the molecular mechanisms of the poplar response to viral infection were reported. The results showed that BCMV infection decreased the chlorophyll content, inhibited the net photosynthesis rate (Pn) and stomatal conductance (Gs), and significantly changed chlorophyll fluorescence parameters in diseased leaves. Transcriptome analysis revealed that the expression of the majority of DEGs (differentially expressed genes) involved in the flavonoid biosynthesis pathway was promoted, but the expression of all or almost all DEGs associated with photosynthesis-antenna proteins and the photosynthesis pathway was inhibited in poplar leaves, suggesting that BCMV infection increased the accumulation of flavonoids but decreased photosynthesis in hosts. Gene set enrichment analysis (GSEA) illustrated that viral infection promoted the expression of genes involved in the defense response or plant-pathogen interaction. MicroRNA-seq analysis illustrated that 10 miRNA families were upregulated while 6 families were downregulated in diseased poplar leaves; moreover, miR156, the largest family with the most miRNA members and target genes, was only differentially upregulated in long-period disease (LD) poplar leaves. Integrated transcriptome and miRNA-seq analyses revealed 29 and 145 candidate miRNA−target gene pairs; however, only 17 and 76 pairs, accounting for 2.2% and 3.2% of all DEGs, were authentically negatively regulated in short-period disease (SD) and LD leaves, respectively. Interestingly, 4 miR156/SPL (squamosa promoter-binding-like protein) miRNA−target gene pairs were identified in LD leaves: the miR156 molecules were upregulated, but SPL genes were downregulated. In conclusion, BCMV infection significantly changed transcriptional and posttranscriptional gene expression in poplar leaves, inhibited photosynthesis, increased the accumulation of flavonoids, induced systematic mosaic symptoms, and decreased physiological performance in diseased poplar leaves. This study elucidated the fine-tuned regulation of poplar gene expression by BCMV; moreover, the results also suggested that miR156/SPL modules played important roles in the virus response and development of viral systematic symptoms in plant virus disease.

## 1 Introduction

Viruses are the second major class of plant pathogens after fungi and severely damage the growth and development of plants and the production of agricultural crops, resulting in more than 100 billion field losses every year in agricultural production worldwide (Strange and Scott, 2005). In addition to annual herbaceous plants and crops, viral pathogens can also infect woody perennial plants such as trees. *Populus* are important model species for plant biotechnology and one of the top three major groups of afforestation tree species in the world, and they can also be infected by various viral pathogens. Poplar leaf mosaic disease is the most common viral disease in poplar species and cultivated species, varieties, and clones and is induced by Poplar Mosaic Virus (PMV, genus *Carlavirus*), and it has been reported in almost all poplar cultivation regions in the world (Biddle and Tinsley, 1971a; Brunt et al., 1976; Xiang, 1982; Smith and Campbell, 2004). In addition, some other viral pathogens, such as Tobacco Mosaic Virus (TMV, genus *Tobamovirus*), Cucumber Mosaic Virus (CMV, genus *Cucumovirus*), Rhabdoviruses (RV, genus *Cytorhabdovirus*), Tobacco Rattle Virus (TRV, genus *Tobravirus*), Tobacco Necrosis Virus (TNV, genus *Alphanecrovirus*), Arabis Mosaic Virus (AMV, genus *Nepovirus*) and Tomato Black Ring Virus (TBRV, genus *Nepovirus*), were reported to be associated with poplar diseases (Nienhaus and Castello, 1989; Xiang, 1990). Research has also shown that one virus isolate of the genus *Potyvirus* induces a decline in inoculated poplar (Martin et al., 1982); however, this poplar-virus interaction has been rarely investigated.

Bean common mosaic virus (BCMV) is one of the most common and most destructive viruses in the genus *Potyvirus*, the largest virus genus of land plants, and it infects common beans (*Phaseolus vulgaris* L.) as well as a range of other cultivated and wild legumes (Morales et al., 2003), and yielded losses could be as high as 50-100% of the production (Damayanti et al., 2008; Saqib et al., 2010; Verma and Gupta, 2010). Although some other plants, such as *Cudrania tricuspidate*, were reported as novel hosts of BCMV (Seo et al., 2015), BCMV is known to have a restricted host range outside legume species (Sáiz et al., 1994). In July 2020, a novel poplar yellow−green mosaic disease was found in a *P. alba* var. *pyramidalis* sapling in the experimental field of our laboratory in Beijing, China. In addition to mosaic symptoms on the leaves, growth retardation of leaves and branched wilting were also observed in diseased poplar. The near-complete genome sequence of BCMV was assembled from the transcriptome sequencing data, and BCMV-like virus particles (flexible rod virions, 10-14 nm in width, and 700 nm in length) were observed by electron microscopy, suggesting that this BCMV virus is a novel pathogen for poplars (Li et al., unpublished data). In the above study, the symptoms of poplar disease, ultrastructure of diseased poplar leaves and virions, and phylogenies of BCMV were reported; moreover, the potential roles of infestation of white-striped long-horned beetles (*Batocera lineolata* Chevrolat) and aphid species that accompanied the occurrence of mosaic disease were also discussed in the above study. Obviously, for this novel poplar mosaic disease caused by BCMV on the non-leguminous host, in addition to our brief introduction above, all aspects of poplar BCMV, particularly the physiological, metabolic, pathological, and gene expression responses of poplar are still unknown.

High-throughput sequencing techniques, such as mRNA, small RNA (sRNA), or microRNA (miRNA) sequencing, provide a powerful tool to investigate the global transcriptome changes of plants in response to pathogen infection. Some studies of virus-plant interaction analyses have been performed at the transcriptional level to study both the physiological and metabolic responses to *Potyvirus* infections in plant hosts (Hillung et al., 2012; Martin et al., 2016; Su et al., 2016; Li et al., 2017; Rashid et al., 2022). For example, using microarray assays, Hillung et al. (2012) investigated the transcript profiles of different *Arabidopsis thaliana* ecotypes in response to *Tobacco etch potyvirus* infection. Dynamic transcriptome profiling of BCMV infection in common bean was performed by time-course mRNA sequencing methods, and the results illustrated that pathways related to defense, gene regulation, metabolic processes, and photosynthesis were specifically altered after viral infection (Martin et al., 2016). MicroRNAs (miRNAs) are a type of endogenous, short (21–23 nucleotides) noncoding RNA molecule that mediate gene regulation through RNA silencing at the posttranscriptional level in eukaryotes (Bartel, 2004). Some studies also have shown that miRNAs mediate gene expression in virus-plant interactions (Pérez-Quintero et al., 2010; Yan et al., 2014; Reyes et al., 2016; Jian et al., 2017). For example, the discovered miRNAs belonged to miR166, miR167, miR169, miR395, miR399, miR408, and miR482 families, which are involved in various pathogenicity or resistance processes against mosaic viruses in maize (Ghorbani et al., 2023). MiR156 is the first identified miRNA in plants (Taylor et al., 2014), which targets and regulates the squamosa promoter-binding-like protein (SPL) transcription factors (Cardon et al., 1997). Through the miR156/SPL regulatory module, miR156 plays important roles in plant developmental stage transformation (Qin et al., 2022) and flowering (Cardon et al., 1999; Wu and Poethig, 2006; Qin et al., 2022). In addition, miR156 plays roles in plant response to abiotic stresses (Chao et al., 2017; Kang et al., 2019; Lan et al., 2019; Wang et al., 2019), response or resistance to bacterial and fungal pathogens (Yin et al., 2019; Sun et al., 2022), and systematic symptom formation in plant-virus interaction. For example, one study showed that co-infection of *Nicotiana benthamiana* with Potato x virus (PXV), Potato y virus (PYV), or Plum pox virus (PPV), resulted in the miR156 accumulation and the most severe systemic mosaic symptoms in *Nicotiana benthamiana* (Pacheco et al., 2012). Integrated mRNA and microRNA (miRNA) transcriptome analysis showed that differentially expressed genes and miRNAs associated with pathogen resistance, such as signal transduction of pathogens and hormones, transcriptional reprogramming, pathogenesis-related genes, etc., were identified in BCMV-infected common bean (Li et al., 2017). Then, a comprehensively genome-wide expression of genes and miRNAs in BCMV-infected poplar leaves and an integrated analysis of their expression would benefit the investigation of the pathology of this novel poplar mosaic disease.

Here, to better understand the effects of BCMV infection on mosaic symptoms developing and photosynthesis of poplar leaves, the assays of photosynthesis pigments content, gas-exchange characteristics, chlorophyll fluorescence parameters, and the genome-wide gene expression profiles (at the transcriptional and posttranscriptional level) of poplar leaves are determined. To reveal the gene regulation infected by BCMV, an integrated analysis of genome-wide mRNA sequencing and miRNA sequencing of poplar leaves is conducted in this study. Finally, the expression of mRNAs and target genes of miRNAs induced by BCMV infection are validated in RT−qPCR assays. This study will improve the understanding of the physiology and pathology of poplar BCMV mosaic disease and will provide an experimental basis for the control and management of this novel poplar disease.

## 2 Results

### 2.1 Symptoms, chlorophyll content, gas-exchange and chlorophyll fluorescence parameters of poplar leaves

**Figure 1:**
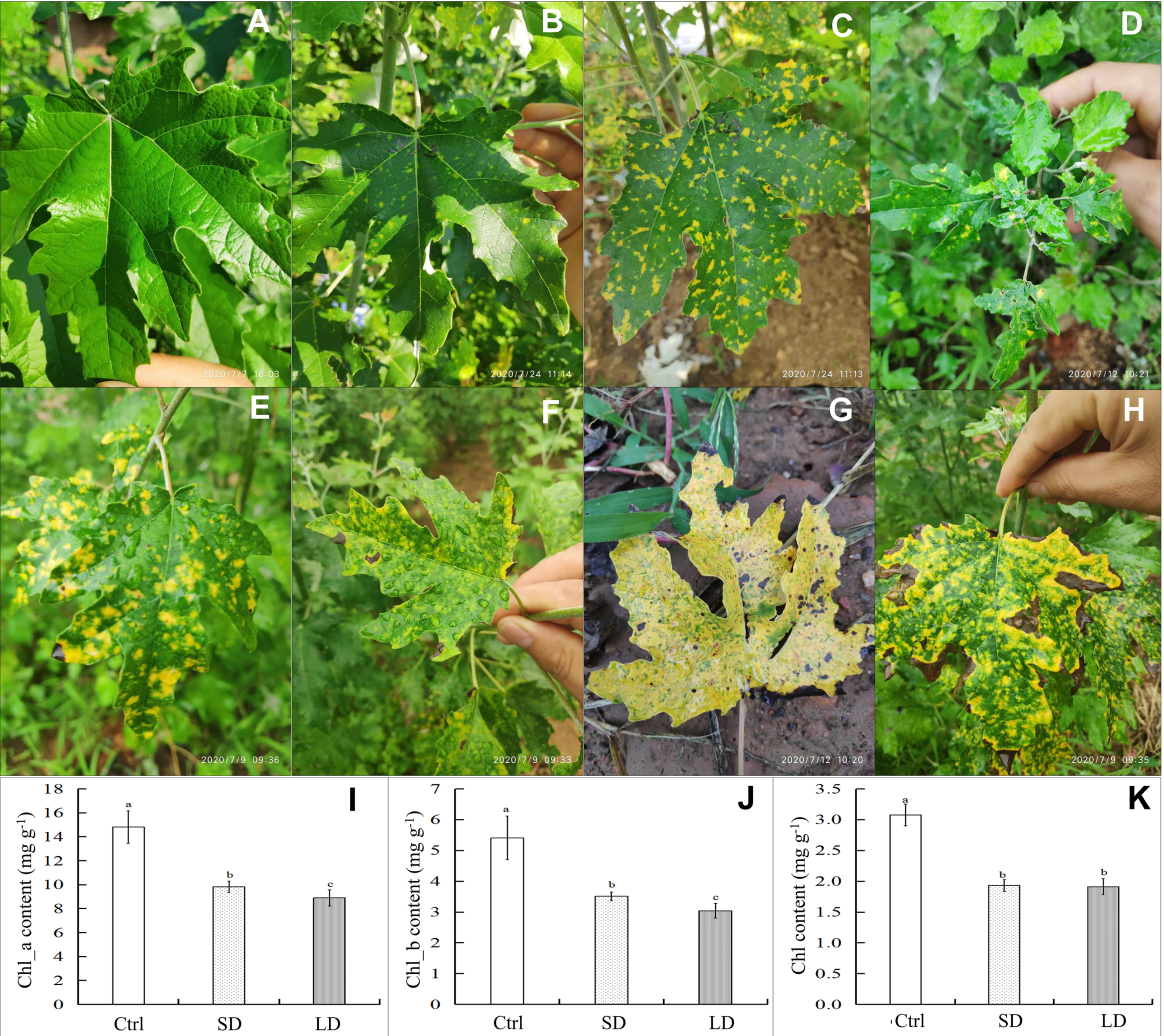
Symptoms and chlorophyll content of leaves in *Populus alba* var. *pyramidalis* infected by BCMV. Representative healthy poplar leaves **(A)**, short-period diseased samples **(B)**, long-period diseased samples **(C)**, mosaic symptoms on immature leaves **(D)**, classic mosaic leaves **(E, F)**, nearly completely chlorotic leaves **(G)**, and edge-withered leaves at the late stage of poplar mosaic disease **(H)**; the content of chlorophyll a (Chl_a) **(I)**, chlorophyll a (Chl_b) **(J)** and total chlorophyll (Chl) **(K)** in poplar leaves. The different letters a, b, or c represent a significant difference in the T-test at the level of p < 0.05, n = 7.

The development of symptoms of poplar mosaic disease was observed on the leaves and branches of poplar saplings in our experimental fields. As shown in **Figure 1**, mosaic symptoms appeared not only in the mature leaves but also in the immature poplar leaves (**Figure 1D**). Mosaic symptoms on poplar leaves were observed at the end of June, with small discrete yellow irregular spots (1-3 mm in diameter) (**Figures 1E, F**). With the development of disease, the diseased leaves became chlorotic or yellow, or the edge of some diseased leaves became curly, darkened, or withered (**Figures 1G, H**). Throughout the entire experiment, healthy poplar leaves remained tender green and did not exhibit mosaic symptoms (**Figure 1A**).

The results showed that mosaic disease significantly decreased the content of photosynthesis pigments (**Figures 1I, K**) in poplar saplings. Specifically, the contents of chlorophyll a (Chl_a), chlorophyll b (Chl_b), and total chlorophyll (Chl) in the healthy leaves were higher than those in the diseased leaves; moreover, the contents of Chl_a and Chl_b in the long-period disease leaves (LD, with classic and significant mosaic symptoms, **Figure 1C**) were lower than those in the short-period disease leaves (SD, with new emerging and slight mosaic symptoms, **Figure 1B**) (T-test, p < 0.05). Chlorophyll is the determinant of green leaf color in plants; therefore, the results of the decrease in chlorophyll content are consistent with the chlorosis symptoms in poplar. However, the yellow coloration that appeared on the diseased leaves was speculated to be the result of the accumulation of flavonoid metabolites.

**Figure 2:**
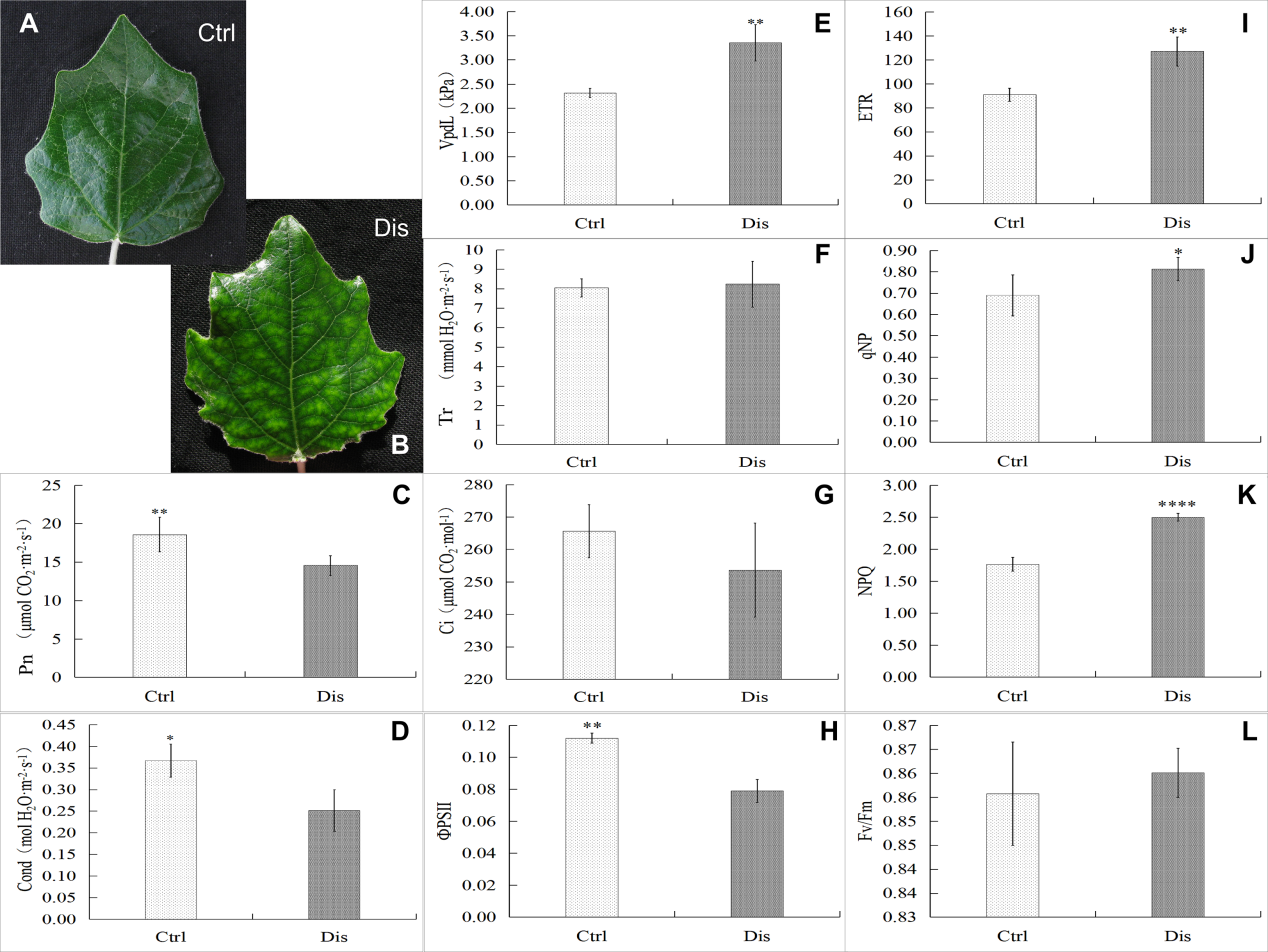
Gas-exchange characteristics and chlorophyll fluorescence parameters in mosaic poplar leaves. Representative healthy poplar leaves (Ctrl, **A**) and diseased samples (Dis, **B**). The gas-exchange characteristics in poplar leaves: the net photosynthesis rate (Pn, **C**), stomatal conductance (Gs, **D**), vapor pressure deficit (VpdL, **E**), transpiration rate (Tr, **F**), and intercellular CO_2_ concentration (Ci, **G**). Chlorophyll fluorescence parameters in poplar leaves: the effective photochemical efficiency of PSII (ΦPSII, **H**), the electron transfer rate (ETR, **I**), the photochemical quenching coefficient (qNP, **J**), the nonphotochemical quenching coefficient (NPQ, **K**) and the maximum photochemical efficiency of PSII (Fv/Fm, **L**). T test, *p < 0.05; **p < 0.01; ****p < 0.001, n = 6.

Gas-exchange characteristic assays revealed that mosaic disease inhibited the net photosynthesis rate (Pn) and stomatal conductance (Gs) and promoted the vapor pressure deficit of leaves (VpdL) but did not influence the transpiration rate (Tr) or intercellular CO_2_ concentration (Ci) in poplar healthy (**Figure 2A**) and diseased (**Figure 2B**) leaf samples (T-test, at least at p < 0.05) (**Figures 2C–G**). To reveal the effects of leaf color changes on photosynthesis, the chlorophyll fluorescence parameters of poplar leaves were detected in this study. The results showed that the effective photochemical efficiency of PSII (ΦPSII) decreased in the diseased leaves (**Figure 2H**), consistent with the decreasing Pn, which also resulted from the decreasing chlorophyll content. In contrast, the values of leaf disease increased the electron transfer rate (ETR), photochemical quenching coefficient (qNP), and nonphotochemical quenching coefficient (NPQ) in the diseased leaves (T-test, at least p < 0.05) (**Figures 2I–K**). However, as a common indicator of abiotic or biotic stresses on plants, the mosaic disease did not change the value of the maximum photochemical efficiency of PSII (Fv/Fm) (**Figure 2L**).

### 2.2 Whole-genome mRNA sequencing reveals gene expression related to poplar BCMV mosaic disease

In this study, Pearson correlation coefficients between samples were used to determine the sample similarity. In mRNA sequencing, in addition to LD3, the coefficient between two samples in the same treatments was higher than 0.80, indicating a high similarity among these samples **(Supplemental Figure 1A**). Therefore, the data of LD3 were excluded from the final analysis of mRNA sequencing. In miRNA sequencing, the coefficient between two samples in the same treatments was all higher than 0.80 (**Supplemental Figure 1B**). Then, all 9 sequencing data of Ctrl, SD, and LD samples in miRNAs sequencing were used in this study.

**Figure 3:**
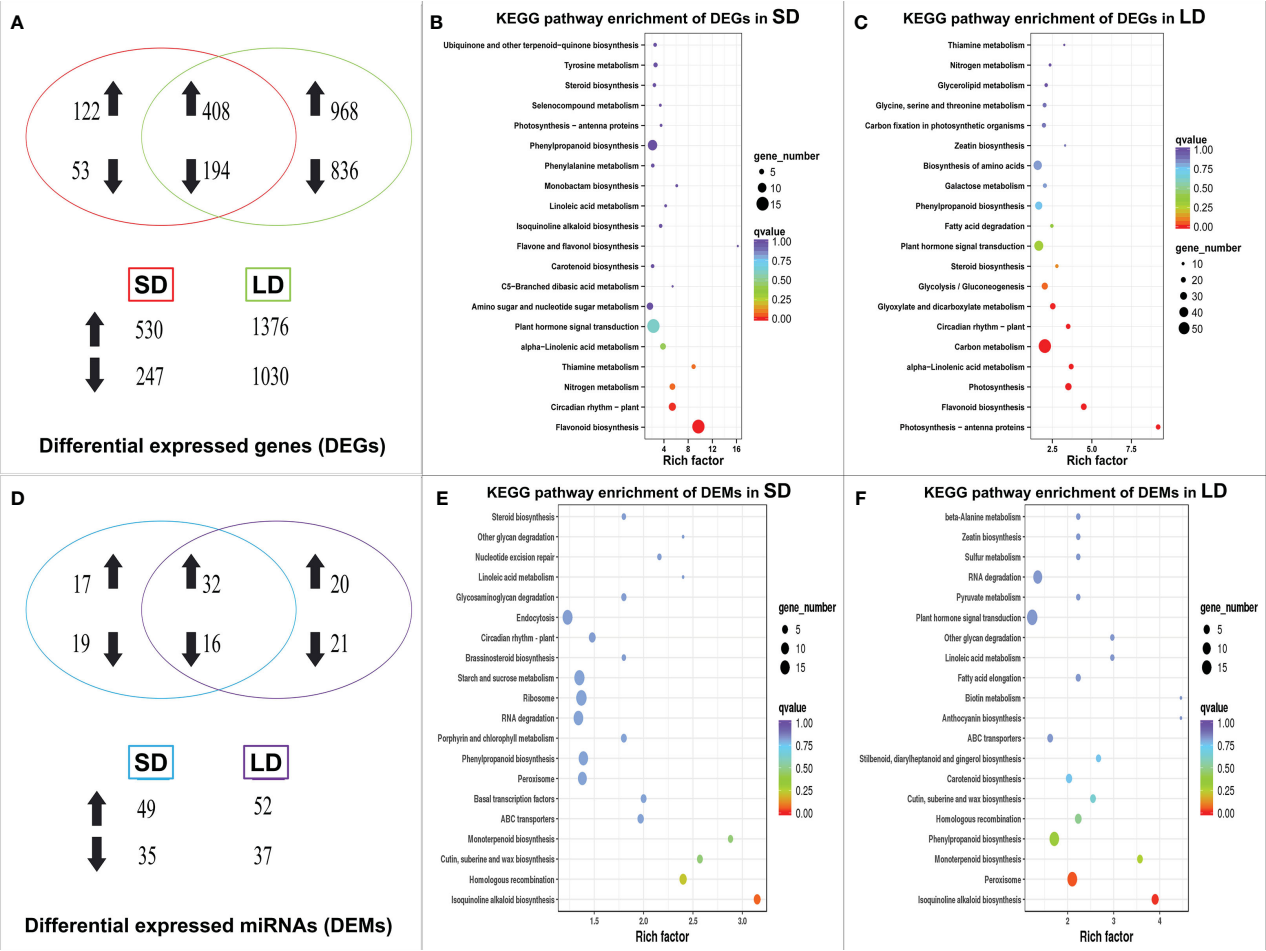
Venn diagram and KEGG pathway enrichment of differentially expressed genes (DEGs) and miRNAs (DEMs) in BCMV diseased poplar leaves, symbolized with up/down arrows. The Venn diagram of DEGs in poplar leaves **(A)**, the statistics of KEGG pathway enrichment of DEGs in SD **(B)** and LD leaves **(C)**; the Venn diagram of DEMs **(D)**, the statistics of KEGG pathway enrichment of DEMs in SD **(E)** and LD leaves **(F)**. SD represented the short-period diseased poplar leaves while LD represented the long-period diseased poplar leaves.

As shown in **Supplemental Table 1**, 777 differentially expressed genes (DEGs) were identified in SD leaves using the mRNA sequencing data, with 530 DEGs upregulated and 247 downregulated; in LD leaves, a total of 2,406 DEGs were identified, 1,376 upregulated and 1,030 downregulated. Moreover, 194 DEGs were consistently downregulated in both SD and LD leaves, while 408 DEGs were upregulated in the two kinds of diseased leaves (**Figure 3A**).

KEGG (Kyoto Encyclopedia of Genes and Genomes) enrichment analysis (Kanehisa and Goto, 2000) was used to identify the significantly changed pathways in mosaic poplar leaves. In SD leaves, flavonoid biosynthesis (KEGG term ko00941) and circadian rhythm-plant (ko04712) were the only two significantly enriched pathways (corrected p-value <0.05) (**Figure 3B**). In LD leaves, photosynthesis-antenna proteins (ko00196) was the most significantly enriched pathway, followed by photosynthesis (ko00195), flavonoid biosynthesis (ko00941), glyoxylate and dicarboxylate metabolism (ko00630), carbon metabolism (ko01200), alpha-linolenic acid metabolism (ko00592), circadian rhythm-plant (ko04712), plant hormone signal transduction (ko04075) and 12 other pathways (corrected p-value <0.05) (**Figure 3C**).

To further reveal the changes in gene expression in leaves after BCMV infection, we compared transcriptome data of leaves experiencing SD and LD treatment (SD was used as a control). As shown in **Supplemental Table 1**, 856 DEGs were identified in SD vs LD leaves, with 567 DEGs upregulated and 289 downregulated. KEGG analysis showed that DEGs involved in flavonoid biosynthesis (ko00941) and plant hormone signal transduction (ko04075) pathway were significantly enriched (corrected p-value <0.05) (**Supplemental Figure 2A**).

#### 2.2.1 Genes involved in the flavonoid biosynthesis pathway

**Figure 4:**
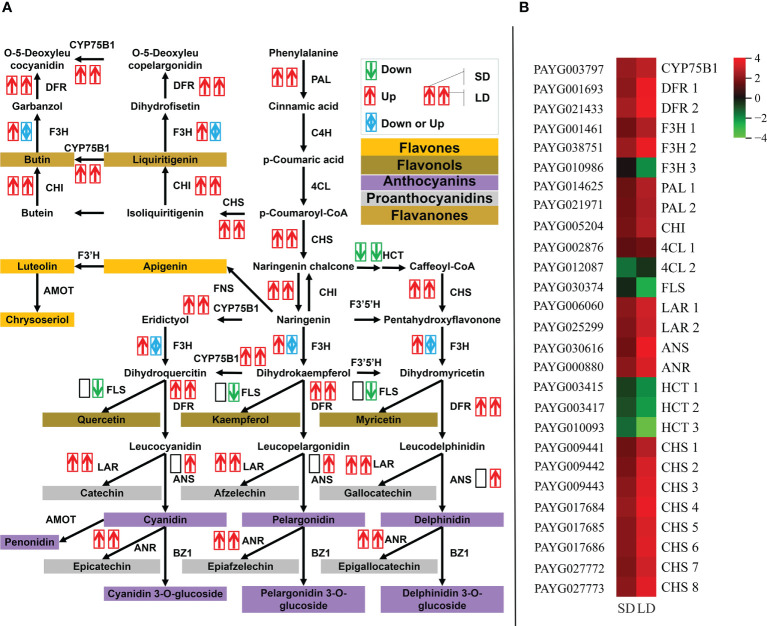
BCMV infection significantly promoted the expression of genes involved in the flavonoid biosynthesis pathway throughout poplar mosaic disease progression. The expression of differentially expressed genes (DEGs) involved in the flavonoid biosynthesis pathway, from phenylalanine to flavonoid metabolites **(A)**, and the expression heatmap of DEGs involved in the flavonoid biosynthesis pathway **(B)**. The red and upward arrow represented the upregulatory expression of genes in poplar leaves, the green and downward arrow represented the downregulatory expression, while the blue and bidirectional arrow represented the mixed regulatory expression of genes. Two side-by-side arrows were used to exhibit the expression of every DEG, the first arrow represented the short-period diseased (SD) leaves, while the second arrow represented the long-period diseased (LD) leaves. ANR, anthocyanin reductase; ANS, anthocyanidin synthase; CHI, chalcone isomerase; CHS, chalcone synthase; CYP75B1, flavonoid 3’-monooxygenase; DFR, dihydroflavonol 4-reductase; FLS, flavonol synthase; HCT, shikimate O-hydroxycinnamoyl transferase; LAR, leucoanthocyantin reductase; PAL, phenylalanine ammonia-lyase.

As shown in **Figures 4A, B**, the expression of 10 DEGs involved in the flavonoid biosynthesis pathway was significantly changed throughout poplar mosaic disease progression, both in SD and LD leaves. Specifically, in addition to the genes encoding HCT (shikimate O-hydroxycinnamoyl transferase), genes encoding 7 flavonoid biosynthesis-related enzymes (proteins) were increased in the SD and LD leaves, such as PAL (phenylalanine ammonia-lyase), CHS (chalcone synthase), CHI (chalcone isomerase), CYP75B1 (flavonoid 3’-monooxygenase), LAR (leucoanthocyantin reductase), DFR (dihydroflavonol 4-reductase) and ANR (anthocyanin reductase) encoding genes. Moreover, the expression of the *PAYG030616* gene encoding anthocyanidin synthase (ANS) was promoted and that of the *PAYG030374* gene encoding FLS (flavonol synthase) was inhibited by BCMV infection in LD leaves (**Figure 4B**). As shown in **Figure 4A**, the upregulation of DEGs involved in flavonoid biosynthesis could increase the accumulation of chalcone, flavanones, flavonols, anthocyanins, etc., resulting in yellow-green mosaic symptoms in diseased poplar leaves.

#### 2.2.2 Genes involved in photosynthesis-related processes

**Figure 5:**
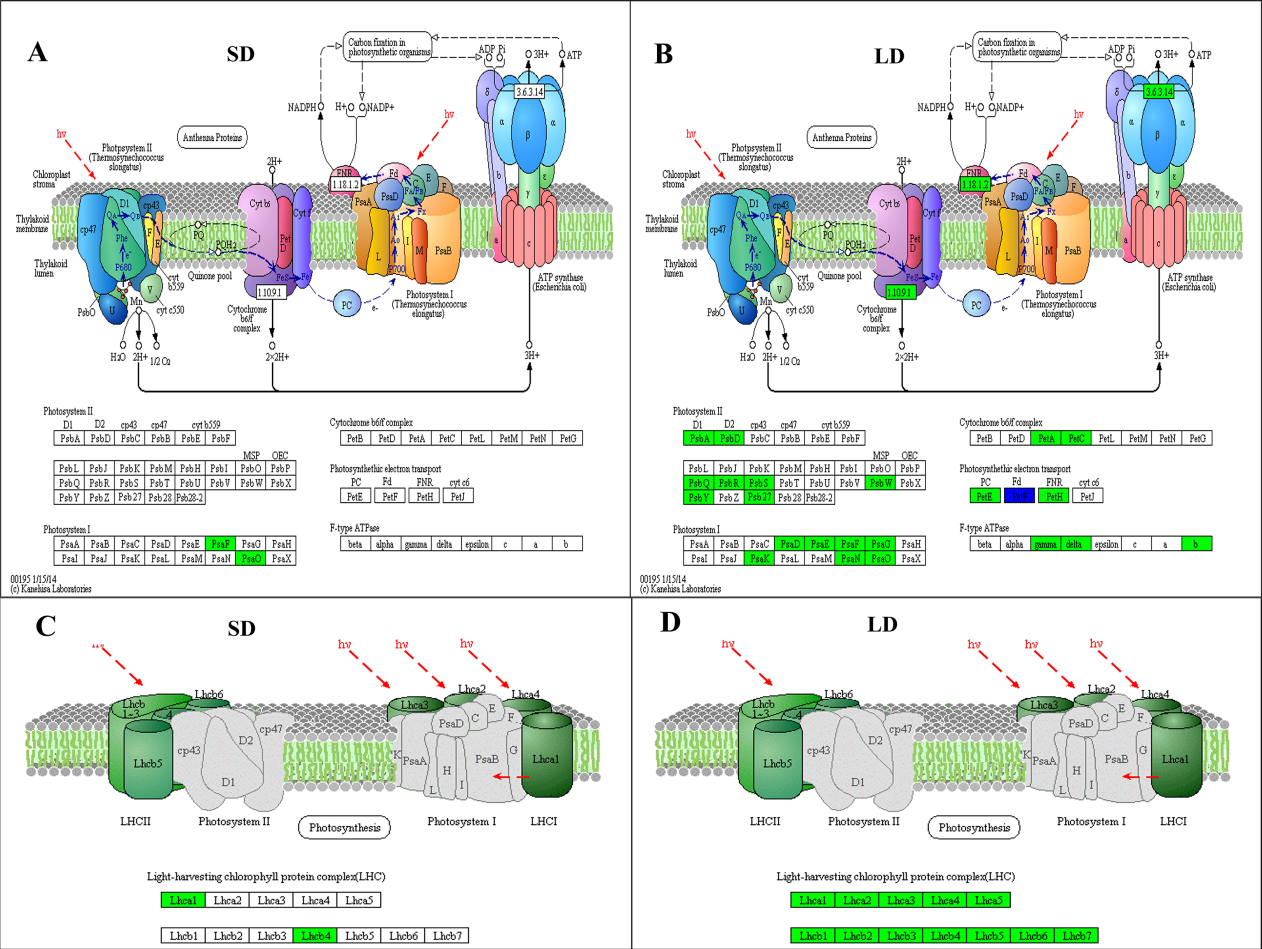
KEGG pathway analysis showed that BCMV infection significantly inhibited the expression of genes involved in photosynthesis and the photosynthetic apparatus in poplar mosaic disease. **(A, B)**, KEGG enrichment results in the Photosynthesis pathway (ko00195); **(C, D)**; KEGG enrichment results in the Photosynthetic apparatus pathway (ko00190). SD: short-period diseased poplar leaves; SD: long-period diseased poplar leaves. Genes in green represent downward adjustment when compared to the control, genes in blue represent both upward and downward adjustment, while no gene was upregulated in SD and LD leaves.

In this study, KEGG enrichment analysis showed that the expression of 2 DEGs (encoding subunits PsaF and PsaO in photosystem I) involved in the photosynthesis pathway was downregulated in SD leaves (**Figure 5A**); however, in addition to the *PAYG008903* gene, the expression of 44 photosynthesis-related DEGs was also downregulated in LD leaves (**Figure 5B**). KEGG annotation showed that the proteins or subunit proteins encoded by these 45 DEGs belonged to all 5 components of photosynthesis: photosystem I, photosystem II, cytochrome b6/f complex, photosynthetic electron transport, and F-type ATPase, therefore, results suggested that BCMV infection inhibited the expression of most DEGs in the photosynthesis pathway (**Figure 5B**). Moreover, the expression of 4 DEGs (encoding subunit proteins Lhca1 and Lhcb4) involved in the photosynthesis-antenna proteins pathway was inhibited in the SD leaves (**Figure 5C**), while the expression of 20 DEGs (encoding 12 subunit proteins Lhca1-5 and Lhcb1-7) was inhibited in the LD leaves (**Figure 5D**). Therefore, these results suggested that viral infection significantly inhibited the whole process of photosynthesis (from light harvesting to carbon fixation) in poplar LD leaves, but did not significantly alter the photosynthesis in SD leaves.

In addition, the results showed that the DEGs in both SD and LD leaves were enriched in the circadian rhythm-plant pathway. In particular, in addition to 8 genes encoding CHS proteins, 10 genes encoding 8 proteins ADO3s (adagio protein 3), PIL1 (phytochrome interacting factor-like 1), PRR95s (pseudo-response regulator 95), CRY2 (cryptochrome-2), PIF3 (phytochrome interacting factor 3), GI (gigantea), RUP2 (repressor of UV-B photomorphogenesis 2) and HD3 (heading date 3) were downregulated in LD leaves (**Supplemental Figure 3**). These results implied that BCMV infection changed the circadian rhythm in poplar trees; however, how viral pathogens change plant rhythm is a question that needs to be further investigated in future studies.

#### 2.2.3 Gene set enrichment analysis reveals that BCMV infection promotes the expression of genes involved in the defense response or plant−pathogen interaction

The results of this study showed that no disease-related pathways or terms were identified as significantly enriched categories in KEGG or GO (Gene Ontology) enrichment analysis based on the DEGs derived from SD and LD samples. To explore the response of gene families with similar biological functions (gene sets) to BCMV infection in poplar, gene set enrichment analysis (GSEA) of all expressed genes in SD and LD leaves compared to healthy leaves was conducted in this study (**Supplemental Table 6**).

**Figure 6:**
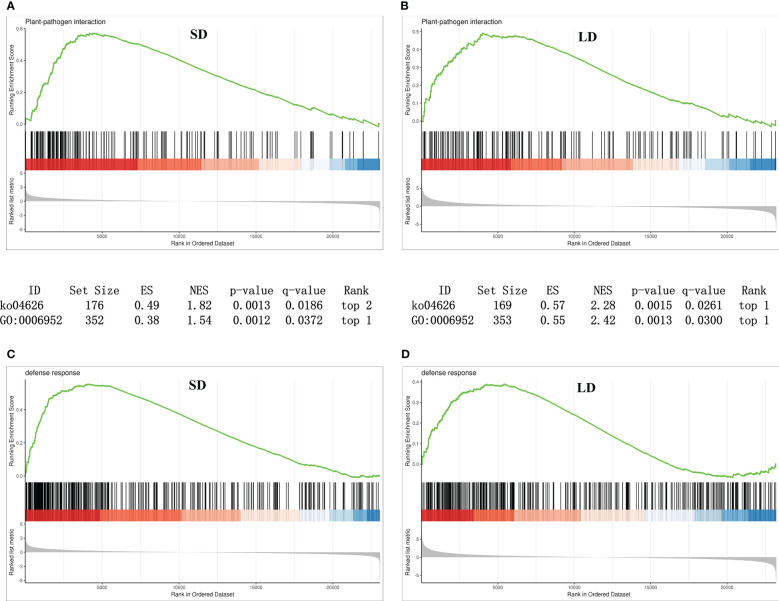
Gene set enrichment analysis (GSEA) revealed that genes involved in plant-pathogen interactions and disease responses were upregulated in BCMV-infected leaves compared to healthy controls. **(A, B)**, GSEA analysis of genes involved in KEGG plant−pathogen interactions pathways; **(C, D)**, GSEA analysis of genes involved in GO defense response term. SD, short-period diseased samples; LD, long-period diseased samples.

As shown in **Figures 6A, C**, in SD leaves, 177 genes involved in the plant−pathogen interaction pathway (KEGG term ko04626) and 352 genes involved in the GO term defense response (GO:0006952 in Biological process) shared upregulated expression patterns; moreover, 169 plant−pathogen interaction-related genes and 353 defense response-related genes shared upregulated expression patterns in LD leaves (**Figures 6B, D**). These results suggested that BCMV infection induced disease resistance in leaves at the early and late stages of poplar mosaic disease.

### 2.3 MicroRNA sequencing reveals gene expression in poplar BCMV mosaic disease

In this study, 330 known and 137 novel mature miRNAs (**Supplemental Table 2**) were identified from nine samples (3 SD, 3 LD, and 3 Ctrl leaves). The known mature miRNAs belonged to 21 miRNA families, and the length distribution of the known miRNAs ranged from 17 to 24 nt. The novel miRNAs ranged from 18-24 nt in length (**Supplemental Figure 4**); however, most of the mature known and novel miRNAs were 21 nt in length.

Expression analysis showed that 78 known miRNAs were differentially expressed in mosaic disease leaves. Specifically, 84 differentially expressed miRNAs (DEMs) were identified in SD (49 upregulated, 35 downregulated), and 89 DEMs (52 upregulated, 37 downregulated) were identified in LD. Among them, 16 miRNAs were continuously downregulated, and 32 miRNAs were continuously upregulated (**Figure 3D**). These 78 DEMs belonged to 21 miRNA families, of which miR156 was the largest family with 12 members, followed by the miR395 family with 10 members and the miR167 family with 6 members. The remaining 18 families contained 2-5 members.

The results also showed that the miRNAs belonging to the same family always shared the same expression pattern in SD and LD leaves; for example, miRNA molecules in the miR1444, miR390, miR397, miR398, miR399, miR408, and miR478 families were upregulated, and miRNA molecules in the miR1446, miR167, miR169, miR394, miR396 and miR350 families were downregulated in the two kinds of diseased leaves. However, 3 miR395 molecules were upregulated in SD leaves, and 7 miR395 molecules were downregulated in LD leaves; 3 molecules of miR482 were divergently expressed in SD leaves, and 2 miR482 molecules were downregulated in LD leaves. In addition, the miR1450, miR160, and miR164 families were only downregulated in SD leaves, and the miR156 (upregulated), miR393 (upregulated), and miR477 (downregulated) families were only expressed in LD leaves. Furthermore, 32 novel miRNAs were identified in SD leaves (8 upregulated, 24 downregulated), and 28 novel miRNAs were identified in LD leaves (16 upregulated, 12 downregulated).

TargetFinder was used to predict the target genes of the known and novel miRNAs. A total of 2,079 target genes were predicted in SD leaves, while 1,656 target genes were predicted in LD leaves (**Supplemental Table 3**). MiR156 was the most abundant known miRNA identified in this study, and 165 target genes were regulated by this molecule, including 13 squamosa promoter-binding-like protein (SPL) genes. In addition, 452 and 337 protein- or transcription factor-encoding genes were predicted as targets of novel_miR_68 and 136, respectively. KEGG pathway enrichment analysis of predicted genes showed that no metabolic pathway was significantly enriched in SD leaves (corrected p value<0.05) (**Figure 3E**). In LD leaves, the most significantly enriched metabolic pathways were peroxisome (ko04146) and isoquinoline alkaloid biosynthesis (ko00950) (p < 0.05) (**Figure 3F**), suggesting that miRNA target genes involved in these two pathways were regulated by miRNAs in a posttranscriptional manner.

Expression analysis showed that 74 known miRNAs were differentially expressed in SD vs LD, of which 31 DEMs were downregulated and 43 DEMs were upregulated. A total of 1,165 target genes were found in SD vs LD (**Supplemental Table 3**). The predicted KEGG pathway enrichment analysis showed that these target genes were significantly enriched in peroxisome (ko04146) and sulfur metabolism (ko00920) (corrected p value<0.05) (**Supplemental Figure 2B**), indicating that the target genes involved in these two pathways are regulated by miRNAs after transcription.

### 2.4 Integrated analysis of mRNA and miRNA sequencing in poplar BCMV mosaic disease

**Figure 7:**
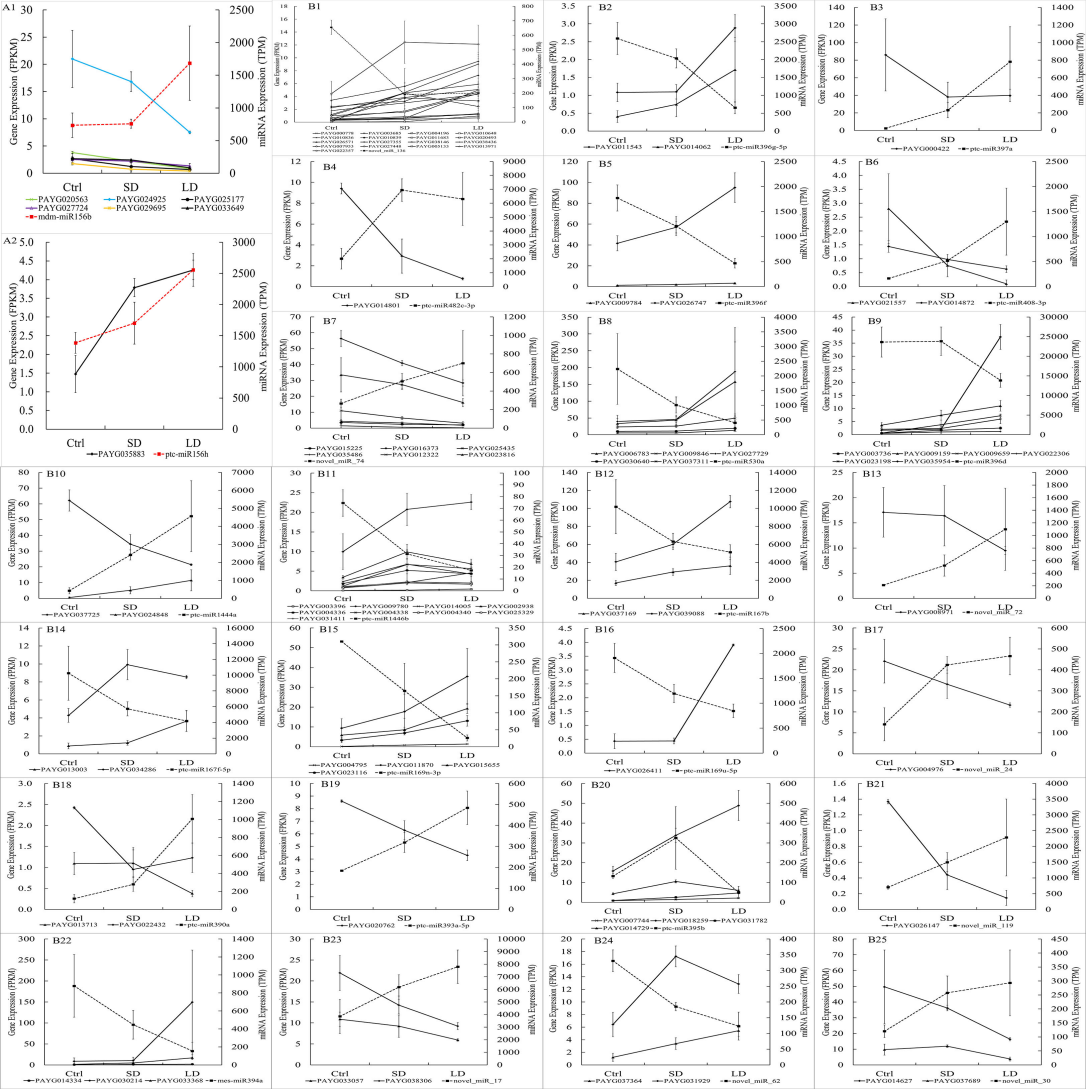
The expression of miRNA and their target genes in poplar leaves infected by BCMV. The x-axis represented samples’ names, the right y-axis represented the TPM expression value of miRNA in miRNA-seq, and the left y-axis represented the FPKM expression value of target genes of miRNA in mRNA-seq. The red or black dotted line represented the expression of miRNAs (in TPM, right y-axis) in miRNA sequencing; the solid line (in black or other colors) represented the expression of their target gene(s) of miRNAs (in FPKM, left y-axis) in transcriptome sequencing. The expression of the miR156 and their targets: A1, expression of 6 target genes, including 4 SPL (squamosa promoter-binding-like protein) gene; A2: expression of target gene *PAYG035883*. The expression of miRNAs and their target gene(s), from B1 to B25, are novel_miR_136, miR396g-5p, miR397a, miR482c-3p, miR396f, miR408-3p, novel_miR_74, miR530a, miR396d, miR1444a, miR1446b, miR167b, novel_miR_72, miR167f-5p, miR169n-3p, novel_miR_24, miR390a, miR393a-5p, miR395b, novel_miR_119, miR394a, novel_miR_17, novel_miR_62, and novel_miR_30, respectively. The expression of miRNAs and target genes were shown as Mean ± SD (n=3).

To validate whether the predicted target genes were regulated by their corresponding miRNAs, an integrated analysis was conducted using the mRNA, miRNA, and predicted target gene expression data. In SD leaves, as shown in **Supplemental Table 4**, a total of 29 candidate miRNA−target pairs (the expression of predicted target genes and their miRNAs both significantly changed) were identified. However, the expression of target genes and miRNAs was inversely changed in only 17 miRNA−target pairs; for example, the expression of ptc-miR395 was downregulated (-1.45, log_2_FC) while the expression of its target gene *PAYG014729* was upregulated (1.11, log_2_FC) in SD leaves. Regarding the negative regulatory role of miRNAs in relation to their targets, this result illustrated that 17 DEGs were regulated by their miRNAs (or regulated by authentic miRNA−target patterns), accounting for 58.62% of all candidate miRNA−target pairs and accounting for only 2.2% of all DEGs in SD leaves. In LD leaves, the expression of 76 DEGs were regulated by their miRNAs, accounting for 52.41% of 145 candidate miRNA−target pairs and 3.2% of all DEGs (**Figure 7**). Then, the integrated analysis of mRNA and miRNA sequencing data illustrated that BCMV infection not only changed the gene expression in the poplar leaves at the transcript level but also finely regulated the gene expression of poplar leaves at the posttranscriptional level.

As mentioned above, the miR156 family is the largest family (with 11 members) and had the most predicted target genes (172 target genes), and all miR156 miRNAs were upregulated in LD leaves. However, as shown in **Supplemental Table 3**, although 18 miR156 predicted target genes were significantly expressed, only 7 genes were the authentic target genes of miR156 for their downregulated patterns in LD leaves. To our surprise, 4 of 7 target genes encoded SPL transcription factors (PAYG024925, PAYG020563, PAYG027724, and PAYG029695) (**Figure 7**). Therefore, our research also suggested that the miR156/SPL module regulated the resistance response to viral pathogens in poplar plants.

### 2.5 Real-Time qPCR validation

**Figure 8:**
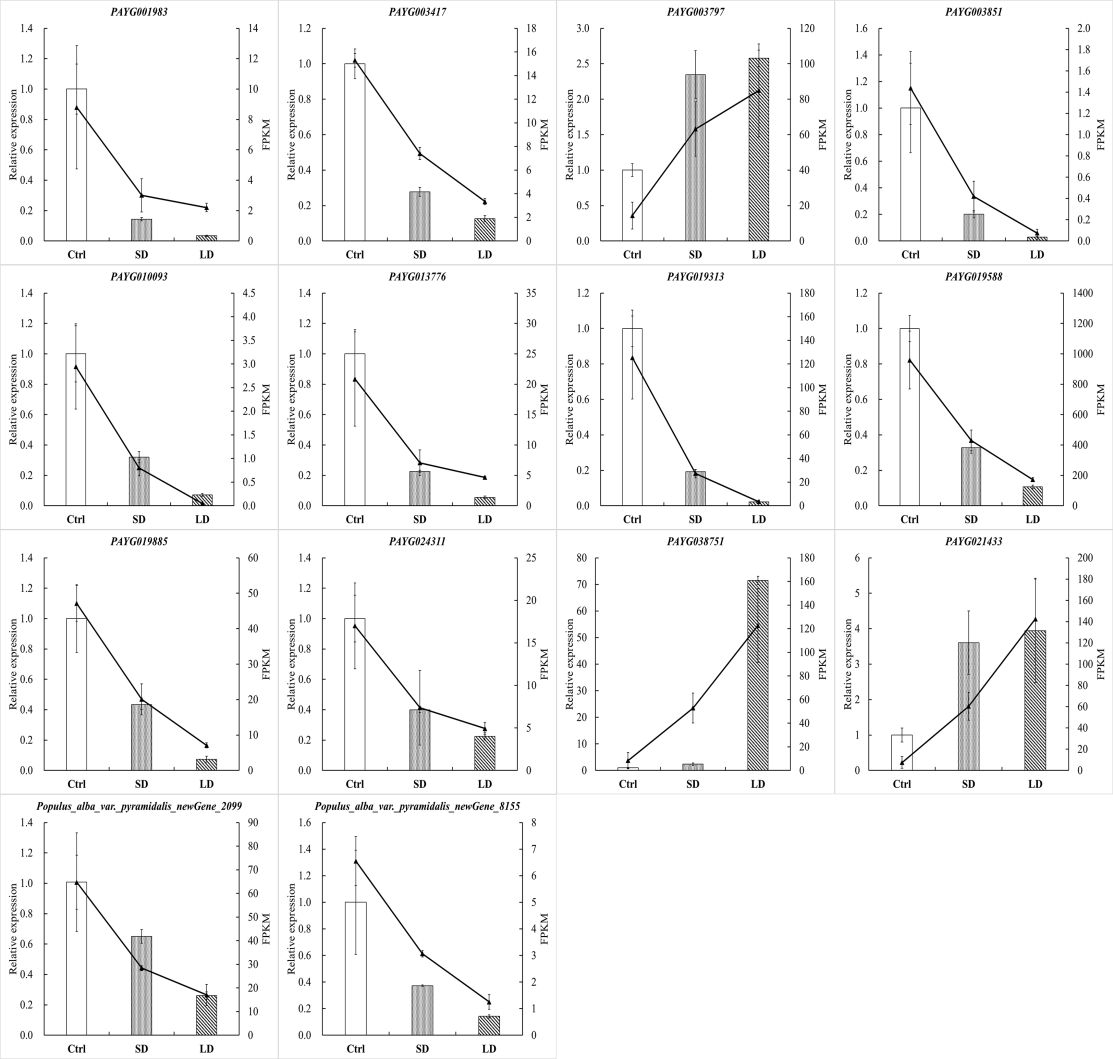
BCMV infection significantly altered gene expression in *P. alba* var. *pyramidalis* leaves. The 2^-ΔΔCt^ was used to calculate the fold change of expression in RT-qPCR analysis, with *EF1β* as a reference for the target gene. All RT−qPCR analyses were performed with four biological and technical replications.

According to global function annotations, 14 DEGs (*PAYG001983*, *PAYG003417*, *PAYG003797*, *PAYG003851, PAYG010093*, *PAYG013776*, *PAYG019313*, *PAYG019588*, *PAYG019885*, *PAYG024311*, *PAYG038751*, *PAYG021433*, *Populus alba* var. *pyramidalis_newGene_2099*, *Populus alba* var. *pyramidalis_newGene_8155*) were manually selected as representatives their potential roles by BCMV infection. As shown in **Figure 8**, the relative expression levels of selected genes were consistent with the transcriptome sequencing results. Although there were some quantitative differences between the two analytical platforms, the similarities between the mRNA-seq data and the real-time qPCR suggested that the mRNA-seq data was reproducible and reliable.

## 3 Discussion

### 3.1 Mosaic and lesion symptoms on poplar leaves induced by BCMV infection

The alternation of color, morphology, and structure of leaves are the most significant and classic symptoms of plant virus diseases, including chlorosis, mosaic, yellowing, spots, stripes, shrinkage, necrosis, etc. (Biddle and Tinsley, 1971b; Ullah et al., 2021). Chlorophyll, flavonoids, anthocyanins, and carotenoids are the main colorants of plants, and chlorophyll and its content determine the color of young and mature leaves. Studies have illustrated that viral infection decreases the content of chlorophyll, destroys the structure and morphology of chloroplasts, and causes mosaic and even yellowing leaves in diseased plants (Tu et al., 1968; Ehara and Misawa, 1975; Goodman and Robert, 1986; Pompe-Novak et al., 2000). In July 2020, we observed mosaic poplar disease in *P. alba* var. *pyramidalis* saplings in our experimental field, assembled the near-complete genome sequence of BCMV from transcriptome sequencing data, and observed BCMV-like virus particles and ultrastructural destruction of the subcellular structure of poplar leaves by electron microscopy (Li et al., unpublished data). This study showed symptom development in poplar leaves, including small and scattered chlorosis spots in young and mature leaves, expanding yellow spots, yellowing leaves, withered and scorched leaves, and twigs, etc. (**Figures 1B–H**). Furthermore, the decrease in chlorophyll content (**Figures 1I–K**) and destruction of chloroplasts induced chlorosis symptoms in diseased leaves, and these changes were also consistent with metrics of physiological performance, such as gas-exchange and chlorophyll fluorescence parameters (**Figures 2C–L**), and molecular regulation patterns at the transcriptional (**Figures 3A–C**) or posttranscriptional level (**Figures 3D–F**).

In addition to the inhibition of photosynthesis through the destruction of leaves or the ultrastructure of chloroplasts, viral pathogens also influence photosynthesis through gene expression regulation. Studies have revealed that viral infection inhibits the expression of the majority of protein-encoding genes located in the chloroplast membrane or thylakoid membrane (Rodríguez et al., 2012; Wu et al., 2013), which is also associated with mosaic symptoms on leaves. Extensive studies have also illustrated that viral pathogens inhibit the expression of chloroplast photosynthesis-related genes (CPRGs), such as the genes encoding the light-harvesting chlorophyll protein complex (LHC), oxygen-evolving complex (OEC) and PSII subunit protein PsbA (Lehto et al., 2003; Pérez-Bueno et al., 2004; Liu et al., 2014; Wang et al., 2015; Ling et al., 2018). Our results in this study are consistent with those of previous studies. The expression of genes involved in photosynthesis and the photosynthesis-antenna protein pathway was inhibited in SD and LD leaves, specifically, at least 2 subunit protein genes of every reaction center of photosynthesis, and all subunit protein genes of LHC were inhibited in LD leaves (**Figures 5A-D**). Therefore, the inhibition of CPRGs induced by the BCMV pathogen plays important roles in the color of diseased leaves.

Flavonoids are the most abundant polyphenol secondary metabolites in plants, including flavones, chalcones, anthocyanins, flavonols, etc. In addition to their roles in resistance to UV light, low temperature, and disease stress (Kootstra, 1994; Torregrosa et al., 2004; Treutter, 2005; Schulz et al., 2016; He et al., 2022), flavonoids are also associated with the coloration of plants (Zhu et al., 2012; Shen et al., 2018; Ni et al., 2020; Stavenga et al., 2020; Zhang et al., 2020). The mosaic symptoms induced by viral pathogens are always related to metabolic disorders of flavonoid biosynthesis. For example, infection with Tobacco Mosaic Virus (TMV) can induce variable mosaic symptoms in tobacco plants, while flavonoid metabolites (such as quercetin and vitexin) can decrease the concentration of virus particles in infected plants and reduce mosaic symptoms at the early stage of infection (Krcatović et al., 2008). Maize leaf infection with Maize Yellow Mosaic Virus (MaYMV) can cause leaf reddening in maize, moreover, upregulated the expression of flavonoid biosynthesis genes, such as chalcone synthase, corresponded with reddening symptoms (Mlotshwa et al., 2021). In this study, pathway enrichment analysis showed that the expression of the majority of DEGs involved in the flavonoid biosynthesis pathway was upregulated before the expression change of genes involved in the photosynthesis-related pathway (**Figures 4A, B** **)**. More than that, compared to chlorophyll, flavonoid pigments accumulated in mesophyll tissue can absorb more high-energy ultraviolet light, which might increase the ETR and qNP in diseased leaves, while high NPQ might reflect abundant photoprotective mechanisms under UV-absorption conditions (Kramer et al., 2004). This result is consistent with symptom change in SD leaves (**Figures 1B–K**) and photoprotection induced by BCMV infection (**Figures 2I–K**), supporting the accumulation of flavonoid metabolites in diseased poplar leaves. Therefore, in addition to the decrease in chlorophyll, the accumulation of flavonoids is suggested as the main reason for mosaic symptoms in BCMV-infected poplar leaves.

Flavonoid metabolites are also associated with plant resistance to UV-B radiation and infections (Torregrosa et al., 2004; Treutter, 2005; Falcone Ferreyra et al., 2012; He et al., 2022). Moreover, some key enzymes related to or involved in the flavonoid biosynthesis pathway, such as PAL (phenylalanine ammonia lyase) (Pant et al., 2021), CHS (chalcone synthase) (Zu et al., 2019), CHI (chalconeisomerase) (Dao et al., 2011), ANR (anthocyanidin reductase) (Su et al., 2022), DFR (dihydroflavonol 4-reductase) and ANR (anthocyanidin reductase) (Kumar et al., 2013), play important roles in the plant response to UV-B radiation and bacterial or fungal infection. High-level expression or overexpression of the genes encoding these enzymes suggested high resistance or tolerance to biotic and abiotic stresses. For PAL and CHS, the high level of expression suggested the accumulation of salicylic acid (SA) and that SA induced systematic resistance because they are important components of the SA biosynthesis pathway (Zu et al., 2019; Pant et al., 2021). In addition, studies have also illustrated that PAL plays a significant role in the production of SA in response to Panicum Mosaic Virus (PMV) and its satellite (SPMV) infections (Pant et al., 2021), while CHS is involved in virus-induced gene silencing in flower senescence of *Petunia hybrida* (Chen et al., 2004). Therefore, as shown in **Figure 4A**, the upregulated expression of genes encoding PAL, CHS, CHI, ANR, etc., involved in the flavonoid biosynthesis pathway suggested an accumulation of flavonoids and resistance to BCMV infection. Flavonol synthase (FLS) catalyzes the oxidation of dihydroflavonol to produce flavonol. In anthocyanin biosynthesis, DFR competes for the substrate (dihydroflavonols) with FLS and reduces dihydroflavonol to its corresponding leucoanthocyanidin. Therefore, the inhibition of FLS genes but the promotion of DFR genes (**Figure 4A**) implied more products of anthocyanins and proanthocyanidins than flavonols. Moreover, aside from gene FLS, the expression of genes encoding PAL, CHS, CHI, F3H, DFR, ANS, and ANR were upregulated in SD and LD leaves, suggesting a *de novo* biosynthesis of flavonoids in BCMV-infected poplar leaves.

Virus infection always altered the endogenous hormone level in host cells, for example, increasing IAA and ethylene content (Van Loon, 1979; Roggero and Pennazio, 1988; Jameson and Sean, 2002), concentration and activity of IAA in host plants (Fraser and Whenham, 1982). Moreover, researches showed that the biological content of ethylene was linearly related to the rate of plaque development and the degree of necrosis (Roggero and Pennazio, 1988; Jameson and Sean, 2002). In this study, KEGG analysis enriched DEGs into plant hormone signal transduction pathway (ko04075). The expression of four AUX/IAA family protein (the negative regulatory factor of auxin biosynthesis) was inhibited in LD leaves when compared to SD leaves, while the expression of five auxin response factor/protein were promoted (**Supplemental Table 1**), suggesting BCMV infection increase the auxin level at the late stage of poplar mosaic disease. In addition, one ethylene response factor gene (*PAYG036981*) was also found to be upregulated in LD leaves. Then, our results validated the dynamic process of hormone levels in plant-virus interactions (Pan et al., 2021), and the changes in auxin and ethylene might associate with the development of mosaic symptoms in poplar leaves.

### 3.2 Virus pathogen-induced photosynthesis-related resistance in poplar leaves

Photosynthesis, a basic and the most important biological process in plants, is also related to plant resistance to biotic and abiotic stresses. Some photosynthesis-related genes are involved in plant resistance to viral pathogens. For example, genes encoding PSII subunit proteins respond to viral infection (Jang et al., 2013); overexpression of the PSII subunit PsbP enhances resistance to AlMV (Alfalfa Mosaic Virus) in *Arabidopsis thaliana* (Balasubramaniam et al., 2014), and silencing of the PsbP gene enhances the symptoms, severity of disease and accumulation of viral particles of RSV (Rice Stripe Virus) in rice and tobacco (Kong et al., 2014). In addition, compared to healthy plants, virus-infected plants always show decreased contents of PsbP and PsbQ (Pérez-Bueno et al., 2004). Ifuku et al. (2005) illustrated that there were no significant physiological differences in low-expression PsbQ-transformed plants compared to wild-type plants; however, growth retardation, chlorosis, or light-green phenotype symptoms and photosynthesis inhibition were observed in low-expression PsbP-transformed tobacco plants. Our results are consistent with previous studies. In addition to the PsbQ gene, the genes encoding PsbR, PsbS, PsbW, PsbY, and Psb27 were also inhibited in LD leaves (**Figure 5B**), suggesting that these genes are also associated with disease resistance or symptom determination in poplar plants.

### 3.3 Posttranscriptional regulation induced by the BCMV pathogen

MicroRNAs (miRNAs) are kinds of short and non-coding RNA molecules, that endogenously produce and negatively regulate the gene expression in the cells of all eukaryotes organisms (Bartel, 2004). In addition to being involved in growth and developmental regulation in plants, miRNAs also play key roles in the response to abiotic stresses and defense against pathogens (Zhang et al., 2006; Zhao et al., 2012). Extensive studies have shown that miRNAs mediate gene expression in virus-plant interactions (Pérez-Quintero et al., 2010; Yan et al., 2014; Reyes et al., 2016; Jian et al., 2017). Some miRNAs, for example, the members in miR166, miR167, miR169, miR395, miR399, miR408, and miR482 families, were involved in the pathogenicity or resistance processes against viruses (Ghorbani et al., 2023). In this study, members of the miR169 family were downregulated, while members of the miR399 and miR408 families were upregulated in SD and LD leaves. MiR395 targeted 27 genes related to energy metabolism and ubiquitination degradation (**Supplemental Table 3**), therefore, the divergently expressed pattern (upregulated in SD leaves but downregulated in LD leaves) might associate with the leaf senescence, programmed death, or the development mosaic symptoms; however, this speculate need further validations.

MiR156 is the largest family identified in this study, 12 members of miR156 were differentially expressed in LD leaves when compared to the control samples. MiR156 targets and regulates the squamosa promoter-binding-like protein (SPL) transcription factors (Cardon et al., 1997) through transcriptional cleavage or translation inhibition (Rhoades et al., 2002). In addition to the main roles in plant phase change and flowering (Cardon et al., 1999; Wu and Poethig, 2006; Xu et al., 2016; Qin et al., 2022), the miR156/SPL module associated with the biosynthesis of flavonoids/anthocyanins (Solangaarachchi and Gould, 2001; Pacheco et al., 2012; Lei et al., 2016; Wang et al., 2019; Qin et al., 2022). The R2R3-MYB transcription factors (MYB113, MYB7) are key regulatory factors in anthocyanin biosynthesis (Cavagnaro et al., 2014; Xu et al., 2019; Chialva et al., 2021; Feng et al., 2021). However, in most cases, MYB-bHLH-WD40 transcriptional activation complex worked together (consistently up- or downregulated) to regulate the expression of anthocyanin biosynthesis structural genes (Winkel-Shirley, 2001; Zhu et al., 2015; Kodama et al., 2018). However, the miR156-targeted SPL transcription factors negatively regulate anthocyanin accumulation by directly preventing the expression of anthocyanin biosynthetic genes through destabilization of the MYB-bHLH-WD40 complex (Gou et al., 2011). Therefore, the miR156 overexpressed in *Arabidopsis*, poplar and tobacco (Gou et al., 2011; Pacheco et al., 2012; Wang et al., 2019) inhibited SPL and resulted in the accumulation of flavonoid/anthocyanin metabolites. In this study, as shown in **Supplemental Table 3**, 7 authentic target genes of the upregulated miR156 were identified in LD leaves when compared to the control, interestingly, 4 of them encoding SPL transcription factors (*PAYG024925*, *PAYG020563*, *PAYG027724*, and *PAYG029695*). Corresponding to the inhibition of 4 SPL genes, most genes involved in the anthocyanins biosynthesis pathway, especially, genes encoding CHS, CHI, DFR, ANS, and ANR, were upregulated in SD/LD leaves when compared to the control samples (**Figure 4A**). Moreover, the regulation of anthocyanins biosynthesis structural genes was consistent with the development of mosaic symptoms in the SD and LD leaves (**Figures 1B, C**). Therefore, this study illustrated BCMV-induced systematic symptoms (classic mosaic symptoms in the whole poplar saplings) through miR156/SPL modules in *P. alba* var. *pyramidalis*. Further, when compared to SD leaves, the expression of genes *PAYG024925* and *PAYG020563* were downregulated in LD leaves (**Supplemental Table 1**) suggesting that these 2 SPL genes play much more roles in the formation and development of systematic symptoms in poplar-BCMV interaction. Considering the roles of flavonoids/anthocyanins in plant resistance, this research suggested that the miR156/SPL module regulated the resistance response to viral pathogens in poplar plants, and played important roles in the development of mosaic disease.

In addition, genomic RNA of viral pathogens can be introduced into host cells and produce double-stranded RNAs (dsRNAs), which are then identified and randomly split into 18-25 nt RNA and virus-derived small RNAs (vsRNAs) by host cell Dicer enzymes (Burgyán and Havelda, 2011). In plant−virus interactions, vsRNA molecules can be sequenced and assembled from the high-throughput sequencing data of host tissues; for example, a novel *Potyvirus* was assembled from pecan trees using a small RNA profile (Su et al., 2016). In our other study, the near-completed genome sequence of BCMV was assembled from the mRNA sequencing data of poplar mosaic leaves (Li et al., unpublished data), and in this study, more than 10 M clean reads (18-30 nt in length) were derived from every miRNA sequencing dataset. However, no BCMV genome sequence or fragments but other viruses (Wuhan insect virus, Drosophila C virus strain EB) were obtained from the miRNA sequencing data of mosaic poplar leaves (data not shown). This result suggested a relatively low number of BCMV-derived small RNAs or a low titer of BCMV in poplar mosaic leaves.

In conclusion, this study reported the physiological performance and gene expression regulation patterns of a novel BCMV mosaic disease in *P. alba* var. *pyramidalis*. BCMV infection significantly decreased the content of total chlorophyll and chlorophyll a and b and inhibited photosynthesis and the effective photochemical efficiency of PSII in diseased poplar leaves. Moreover, BCMV infection induced significant changes in gene expression at both the transcriptional and posttranscriptional levels. Flavonoid biosynthesis- and photosynthesis-related pathways were enriched in this study, accounting for the symptoms and physiological performance induced by a viral infection and revealing the host responses to the pathogen. Many genes and miRNAs were differentially expressed in the diseased poplar leaves; however, only 2.2 to 3.2% of all DEGs were authentically regulated by their corresponding miRNAs, suggesting fine-tuning of posttranscriptional regulation patterns. This study revealed 4 miR156/SPL modules in BCMV-infected poplar leaves. To the best of our knowledge, this is the first report of miR156/SPL modules involved in plant viral disease.

## 4 Materials and methods

### 4.1 Plant materials and experiments

Approximately 50 P*. alba* var. *pyramidalis* saplings were collected from a poplar plantation in Linhe County (Inner Mongolia, China) in April 2017 and cultivated in the experimental field of our laboratory at the Chinese Academy of Forestry in Beijing, China. The saplings were trimmed at the branches 30 cm above their base every year to keep a four-year-old root and one-year-old branches. Poplar saplings were well irrigated and regularly subjected to pest control to maintain the health of plants. However, typical leaf mosaic symptoms were observed on the branches that sprouted from one side of the basal branch of one sapling on 12^th^ July 2020; approximately 15-20 days later, mosaic symptoms appeared on the leaves of the branches that sprouted from the other side. Then, three 6-7^th^ mature leaves from the top of different branches on the long-period diseased (named LD leaves, with classic and significant mosaic symptoms) side and short-period diseased (SD leaves, with new emerging and slight mosaic symptoms) side were collected to extract total RNA. Three control leaves were collected from three healthy poplars (without any mosaic or decline symptoms), and all leaves were 6-7^th^ mature leaves and were identical to LD and SD leaves in size and location on the poplar trees.

### 4.2 Determination of photosynthesis physiology

In this study, to reveal the effect of viral infection on poplar leaves, the gas-exchange characteristics, chlorophyll fluorescence parameters, and chlorophyll content were determined using a Li-6400XT portable photosynthesis system (LI-COR, Lincoln, USA) (Xing et al., 2020; Xing et al., 2022). In the gas-exchange assays, the net photosynthesis rate (Pn), stomatal conductance (Gs), intercellular CO_2_ concentration (Ci), transpiration rate (Tr), and vapor pressure deficit of leaves (VpdL) were determined. The chlorophyll fluorescence parameters were determined using a Fluorescence monitoring System (FMS, Hansatech, British). In the chlorophyll fluorescence parameter assays, the maximum photochemical efficiency of PSII (Fv/Fm), electron transfer rate (ETR), photochemical quenching coefficient (qNP), and nonphotochemical quenching coefficient (NPQ) were calculated. Seven healthy mature leaves and seven leaves with mild mosaic symptoms were detected in the gas-exchange and chlorophyll fluorescence assays. In this study, approximately 0.2g leaf tissue collected from 6 mature leaves (Ctrl, SD, and LD samples) was used in the determination of chlorophyll content, chlorophyll a (Chl_a), chlorophyll b (Chl_b) and total chlorophyll (Chl) (Xing et al., 2020; Xing et al., 2022).

### 4.3 Total RNA extraction, cDNA library construction, and mRNA and miRNA sequencing

Total RNA was extracted with TRIzol, and RNA samples with good quality (A_260_/A_280_ between 1.9–2.1, RIN (RNA integrity number) > 7) were used to construct libraries for high-throughput sequencing and PCR amplification. Briefly, mRNA was isolated from total RNA using oligo(dT) magnetic beads, and then mRNA was fragmented randomly. According to the mRNA sequence, the first strand of cDNA was synthesized using oligo(dT) primers, and then the second strand of cDNA was synthesized. Transcriptome libraries were generated using the NEBNext^®^Ultra™ RNA Library Prep Kit for Illumina (NEB, USA) following the manufacturer’s recommendations. The cDNA libraries of virus-infected samples were sequenced using the Illumina NextSeq 2000 platform (Illumina, San Diego, CA), and paired-end reads were generated. Clean reads were obtained by discarding reads containing adapters, reads containing poly-Ns and low-quality reads. The 9 clean transcriptome datasets of poplar were deposited in the NCBI database. After removing the low-quality (Q ≤ 20) and adapter reads, the clean reads were assembled according to the reference genome sequence data with HISATv2.1.0. The *P. alba* var. *pyramidalis* genome sequence v1.0 (https://bigd.big.ac.cn/search/?dbId=gwh&q=GWHAAEP00000000) was used in this study as the reference genome. Gene expression was measured in fragments per kilobase of transcript per million fragments mapped (FPKM). DESeq2 was used to identify the differentially expressed genes (DEGs) between the two groups, with FDR < 0.05 and |log_2_FC| > 1, or for unique genes, FDR < 0.05, used as the threshold for DEGs. Finally, the annotation of predicted target genes was conducted using the NCBI, Swiss-Prot, COG, KEGG, KOG, and Pfam databases.

For miRNA libraries, 18-28 nt miRNAs were sequentially ligated to a 3′ adapter and a 5′ accepter and purified using 15% denaturing PAGE electrophoresis. The final purified ligation products were reverse transcribed into cDNA. The first strand of cDNA was PCR amplified, DNA amplicons from each library were purified and separately submitted for high-throughput sequencing on the Illumina platform, and single-end reads were generated. Clean data (clean reads) were obtained by removing reads containing adapters, reads containing poly-Ns and low-quality reads from raw data, and clean reads were trimmed and cleaned by removing sequences smaller than 18 nt or longer than 30 nt. The 9 clean sRNA sequencing datasets were deposited in the NCBI database. In this study, known miRNAs were identified in the miRNA database miRBsse v22. Compared to known miRNAs, only 1 mismatch nucleotide in the identified mature sequence of miRNAs was permitted, while MiRDeep2 (parameters: -g -1 -b 0) was used to identify novel miRNAs (Friedländer et al., 2012; Zhang et al., 2015). The expression of miRNAs was measured in transcripts per million mapped reads (TPM). DESeq2 was used to identify the differentially expressed miRNAs (DEMs) between the two groups, with FDR < 0.05 and |log_2_FC| >1, or for unique genes, FDR < 0.05, used as the threshold for DEGs. The target genes of known and novel miRNAs were predicted using TargetFinder (parameters: -c 3) based on the mature sequence of miRNAs and poplar genome sequence (https://github.com/carringtonlab/TargetFinder). Finally, the annotation of predicted target genes was conducted using the NCBI, Swiss-Prot, COG, KEGG, KOG, and Pfam databases.

### 4.4 Enrichment analysis

In this study, the DEGs and miRNA target genes were used in enrichment analysis. The enrichment analysis was performed at the level of KEGG metabolism pathways or the biological process (BP), molecular function (MF), and cellular component (CP) terms in the GO (Gene Ontology) database.

Gene set enrichment analysis (GSEA) (Subramanian et al., 2005) is commonly used for enrichment analysis of all genes based on the expression of all genes without prior knowledge. In contrast to the general enrichment analysis based on DEGs, GSEA can uncover the weak but consistent expression trends of gene sets (a cluster of genes, always 10-1000 genes, involved in the specific physiological process or pathway with similar functions or any genes of interest) that are not significantly differentially expressed. Combined with physiological, epigenetic, and molecular characteristics, GSEA can uncover the biological significance of gene sets without a differential expression threshold. The GSEA in this study was also performed using the KEGG and GO databases. Finally, a p-value < 0.001 and an FDR < 0.05 were used as the criteria for the significantly enriched gene set.

### 4.5 Integrated analysis of mRNA and miRNA sequencing data

To reveal the genes regulated by their miRNAs in poplar leaves, the expression data of each miRNA predicted target gene were derived from DEG data of the SD vs. Ctrl or LD vs. Ctrl comparison and then compared with the expression of its miRNAs. If the target gene and its miRNAs were both significantly expressed in the same comparison, the target and the miRNA were named a candidate miRNA−target pair. For the negative regulatory patterns of target expression by the miRNAs, the expression of miRNAs and genes in an authentic miRNA−target pair should be divergently regulated.

### 4.6 RT−qPCR validation

In this study, 14 DEGs closely related to plant energy synthesis and metabolism identified by transcriptome analysis were validated by RT−PCR using a FastKing RT Kit from TIANGEN Co. (Beijing). The RT−qPCR primers were designed using National Center for Biotechnology Information Primer BLAST tools (available online: http://www.ncbi.nlm.nih.gov/tools/primer-blast/). Primer sequences and amplification efficiency are listed in **Supplemental Table 5**. In this study, as in Zhao’s method (Zhao et al., 2017), elongation factor 1-beta (*EF1β, PAYG034648*) was selected as the best reference gene for RT−qPCR validation from transcriptome data of leaves. Relative transcript levels of target genes were calculated using the 2^-ΔΔCt^ formula. All RT−qPCR analyses were performed with four biological and technical replications.

## Data availability statement

The datasets presented in this study can be found in online repositories. The names of the repository/repositories and accession number(s) can be found in the article/**Supplementary Material**.

## Author contributions

JZ planned and designed the research and contributed to the original concept of the manuscript. LW and WZ analyzed all the data. WS, ML, ZL, and YF performed the collection and processing of plant samples. WZ, WS, ML, and JL participated in all experiments. LW and JZ wrote the manuscript. HL and BZ revised the draft manuscript. XS supervised the experiment. All authors contributed to the article and approved the submitted version.

## Supplementary material

Supplementary Figure 1 Sample clustering heat map. **(A)** represents the Pearson correlation coefficient between mRNA-seq samples; **(B)** represents the Pearson correlation coefficient between miRNA-seq samples.

Supplementary Figure 2 KEGG pathway enrichment of differentially expressed genes (DEGs) and miRNAs (DEMs) in SD vs LD. The statistics of KEGG pathway enrichment of DEGs **(A)** and DEMs **(B)**. SD represented the short-period diseased poplar leaves and LD represented the long-period poplar leaves.

Supplementary Figure 3 The expression on the heatmap of DEGs involved in the circadian rhythm-plant pathway.

Supplementary Figure 4 miRNAs length distribution. Known miRNAs length distribution **(A)**, novel miRNAs length distribution **(B)**.
