# Supplementary figures and images for: Integrated transcriptome and microRNA sequencing analyses reveal gene responses in poplar leaves infected by the novel pathogen bean common mosaic virus (BCMV)
Source: Front Plant Sci. 2023 Jun 15;14:1163232. doi: 10.3389/fpls.2023.1163232 (PMC10308444; doi:10.3389/fpls.2023.1163232)

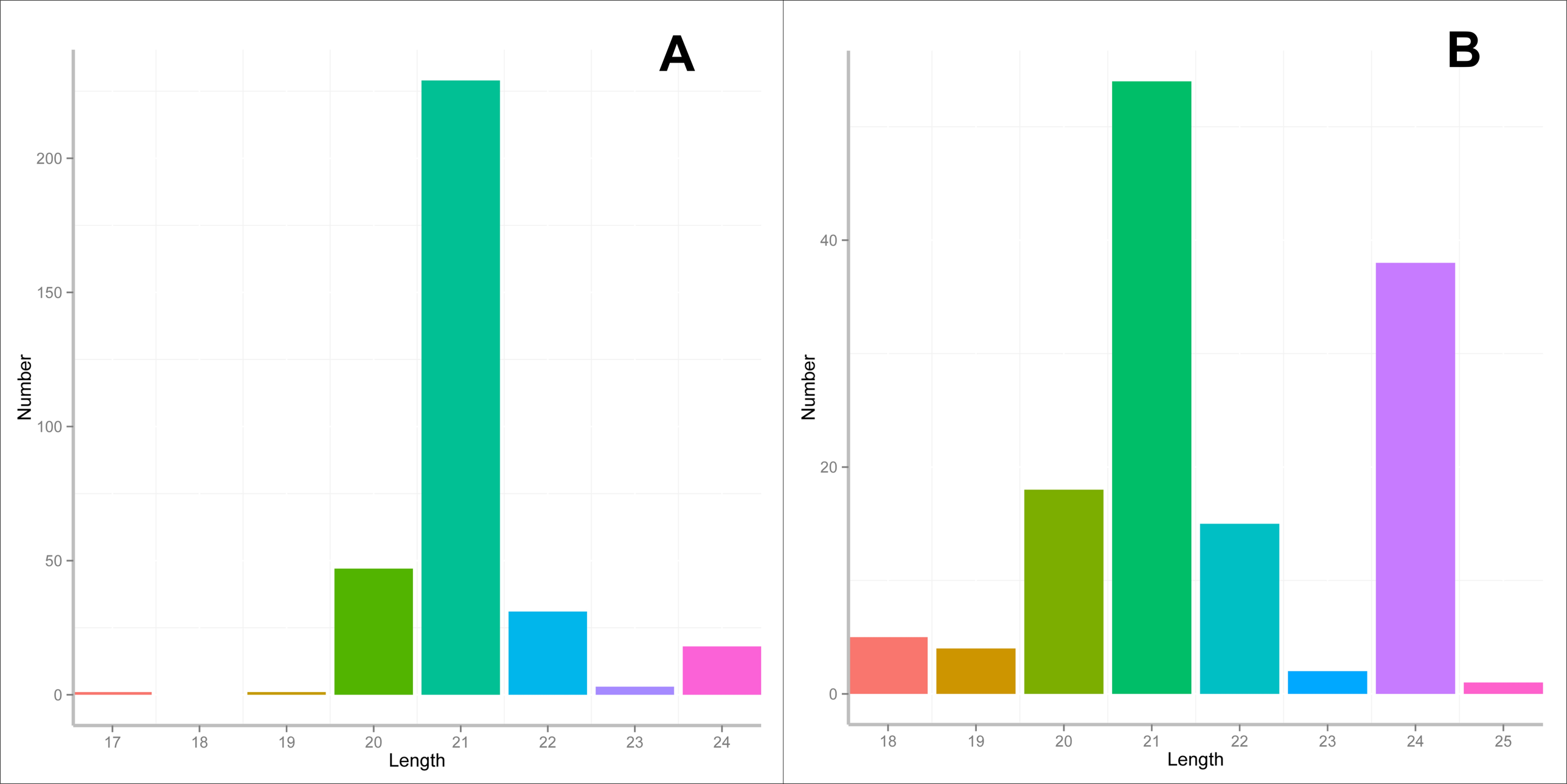

Supplement: Supplementary Figure 4 — miRNAs length distribution. Known miRNAs length distribution (A), novel miRNAs length distribution (B). [file Image_4.jpeg]
